# Impact of different haemodynamic resuscitation strategies on brain perfusion and tissue oedema markers in a model of severe haemorrhagic shock

**DOI:** 10.1186/cc12156

**Published:** 2013-03-19

**Authors:** KK Ida, DA Otsuki, LU Castro, TR Sanches, MH Shimizu, LC Andrade, JO Auler-Jr, LM Malbouisson

**Affiliations:** 1 Faculdade de Medicina, Universidade de São Paulo, Brazil

## Introduction

This study aimed to compare the cerebral effects of terlipressin (TERLI) with conventional prehospital fluid resuscitation with lactated Ringer's solution (LR) in a model of haemorrhagic shock (HS).

## Methods

Pigs (20 to 30 kg) were randomized into one of the groups: Sham (*n = *2), HS (*n = *9), LR (3× volume bled; *n = *9) or TERLI (2 mg bolus; *n = *9). HS induced to target MAP of 40 mmHg was maintained for 30 minutes. Brain tissue oxygen pressure (PbtO_2_), intracranial pressure (ICP), cerebral perfusion pressure (CPP), haemodynamics and blood gas analyses were assessed prior to HS (baseline) up to 120 minutes after treatment. Tissue markers of brain oedema (aquaporin-4 (AQP4) and Na-K-Cl cotransporter-1 (NKCC1)), apoptosis (pre-apoptotic protein (Bax)) and oxidative stress (thiobarbituric acid reactive substances (TBARS)) were also measured.

## Results

Sham animals had no significant changes in the variables assessed. HS resulted in a significant decrease in CPP (mean varied from 36 to 39 mmHg), PbtO_2 _(from 23.6 to 26.6 mmHg), ICP (from 1 to 2 mmHg) and haemodynamics (MAP from 38 to 40 mmHg; CI from 1.8 to 2.1 l/minute/m^2^), and a significant increase in blood lactate (from 6.7 to 8.9 mmol/l) and cerebral AQP4 (mean ± SE; 167 ± 54% of sham), NKCC1 (237 ± 47% of sham), Bax (167 ± 44% of sham) and TBARS. Fluid resuscitation was followed by an increase in ICP (from 7 to 9 mmHg) and a decrease in CPP (from 41 to 52 mmHg), with an increased expression of cerebral AQP4 (210 ± 56% of sham), NKCC1 (163 ± 32% of sham) and Bax (137 ± 24% of sham). Only TERLI restored baseline values of CPP (from 54 to 61 mmHg) and did not change the cerebral expression of AQP4 (100 ± 6% of sham), NKCC1 (100 ± 1% of sham), Bax (102 ± 6% of sham) and TBARS. Both TERLI and LR recovered baseline levels of PbtO_2 _(TERLI from 30.0 to 34.2 mmHg; LR from 29.4 to 40.7 mmHg) and MAP (TERLI from 53 to 64 mmHg; LR: 48 to 61 mmHg). Blood lactate levels were not recovered in any group (TERLI from 5.7 to 8.1 mmol/l; LR from 4.5 to 7.7 mmol/l).

## Conclusion

TERLI recovered cerebral perfusion and oxygenation with no significant changes in ICP and cerebral markers of oedema, apoptosis and oxidative stress. LR did not recover CPP probably due to a significant increase in ICP caused by brain oedema, which may have contributed to the cerebral apoptosis. None of the treatments caused cerebral oxidative stress.
